# Air Pollution and Lung Function in Dutch Children: A Comparison of Exposure Estimates and Associations Based on Land Use Regression and Dispersion Exposure Modeling Approaches

**DOI:** 10.1289/ehp.1408541

**Published:** 2015-04-03

**Authors:** Meng Wang, Ulrike Gehring, Gerard Hoek, Menno Keuken, Sander Jonkers, Rob Beelen, Marloes Eeftens, Dirkje S. Postma, Bert Brunekreef

**Affiliations:** 1 Institute for Risk Assessment Sciences, Utrecht University, Utrecht, the Netherlands; 2 TNO, Netherlands Organisation for Applied Scientific Research, Utrecht, the Netherlands; 3 Swiss Tropical and Public Health Institute, Basel, Switzerland; 4 University of Basel, Basel, Switzerland; 5 University of Groningen, Department of Pulmonology, University Medical Center Groningen, Groningen, the Netherlands; 6 Julius Center for Health Sciences and Primary Care, University Medical Center Utrecht, Utrecht, the Netherlands

## Abstract

**Background:**

There is limited knowledge about the extent to which estimates of air pollution effects on health are affected by the choice for a specific exposure model.

**Objectives:**

We aimed to evaluate the correlation between long-term air pollution exposure estimates using two commonly used exposure modeling techniques [dispersion and land use regression (LUR) models] and, in addition, to compare the estimates of the association between long-term exposure to air pollution and lung function in children using these exposure modeling techniques.

**Methods:**

We used data of 1,058 participants of a Dutch birth cohort study with measured forced expiratory volume in 1 sec (FEV_1_), forced vital capacity (FVC), and peak expiratory flow (PEF) measurements at 8 years of age. For each child, annual average outdoor air pollution exposure [nitrogen dioxide (NO_2_), mass concentration of particulate matter with diameters ≤ 2.5 and ≤ 10 μm (PM_2.5_, PM_10_), and PM_2.5_ soot] was estimated for the current addresses of the participants by a dispersion and a LUR model. Associations between exposures to air pollution and lung function parameters were estimated using linear regression analysis with confounder adjustment.

**Results:**

Correlations between LUR- and dispersion-modeled pollution concentrations were high for NO_2_, PM_2.5_, and PM_2.5_ soot (*R* = 0.86–0.90) but low for PM_10_ (*R* = 0.57). Associations with lung function were similar for air pollutant exposures estimated using LUR and dispersion modeling, except for associations of PM_2.5_ with FEV_1_ and FVC, which were stronger but less precise for exposures based on LUR compared with dispersion model.

**Conclusions:**

Predictions from LUR and dispersion models correlated very well for PM_2.5_, NO_2_, and PM_2.5_ soot but not for PM_10_. Health effect estimates did not depend on the type of model used to estimate exposure in a population of Dutch children.

**Citation:**

Wang M, Gehring U, Hoek G, Keuken M, Jonkers S, Beelen R, Eeftens M, Postma DS, Brunekreef B. 2015. Air pollution and lung function in Dutch children: a comparison of exposure estimates and associations based on land use regression and dispersion exposure modeling approaches. Environ Health Perspect 123:847–851; http://dx.doi.org/10.1289/ehp.1408541

## Introduction

Currently, there is an increased interest in estimating health effects with individual estimates of exposure, taking into account intra-urban differences in air pollution levels (Brauer et al. 2008; Gehring et al. 2013; Mölter et al. 2013), because of potential underestimation of health effects based on exposure assignment at community level (Jerrett et al. 2005b; Miller et al. 2007).

Land use regression (LUR) modeling and dispersion modeling have been extensively applied to characterize small-scale spatial variability of air pollution (Jerrett et al. 2005a). These approaches are based on distinctive methodological principles. LUR modeling combines data from air pollution measurements with data from geographic information systems (GIS) and stochastic modeling that exploits land use, geographic, and traffic characteristics to explain spatial concentration variations at measured sites. Dispersion modeling relies on deterministic (e.g., Gaussian plume) equations and uses data on emission, meteorological conditions, and topographical data to simulate the physicochemical processes of transport and atmospheric chemistry when estimating outdoor air pollution concentrations (Jerrett et al. 2005a). At present, comparisons of the prediction ability of LUR and dispersion models at cohort addresses are scarce (Beelen et al. 2010; Briggs et al. 2000; Cyrys et al. 2005; Dijkema et al. 2011; Gulliver et al. 2011; Marshall et al. 2008).

Recent studies have raised the importance of comparing alternative exposure metrics and relevant health effects in epidemiological studies (Baxter et al. 2013; Özkaynak et al. 2013). The impact of dispersion and LUR modeling on health effect estimates has been investigated only in a California study and a French study on the effects of air pollution on pregnancy outcomes. These studies reported comparable results for the two modeling approaches (Sellier et al. 2014; Wu et al. 2011). However, only models for nitrogen dioxide (NO_2_) and particulate matter with diameter ≤ 10 μm (PM_10_) were compared in these studies.

The aims of this study were *a*) to evaluate the agreement between long-term air pollution exposure estimates for NO_2_, particulate matter with diameter ≤ 2.5 μm (PM_2.5_), PM_2.5_ soot, and PM_10_ based on dispersion modeling and LUR modeling; and *b*) to evaluate whether associations between long-term air pollution exposures and lung function in children differ depending on the exposure modeling approach used.

## Methods

*Study population*. We included participants from the Dutch PIAMA (Prevention and Incidence of Asthma and Mite Allergy) birth cohort study. For the study, pregnant women were recruited in 1996–1997 during their second trimester of pregnancy from a series of areas in the north, west, and center of the Netherlands. Nonallergic pregnant women were invited to participate in a “natural history” study arm. Pregnant women identified as allergic through a validated screening questionnaire were allocated to an intervention arm with a random subset allocated to the natural history arm. The study started with 3,963 newborns. Ethics approval to perform the study was obtained from the local authorized institutional review boards, and written informed consent was obtained from the parents or legal guardians of all participants. More information about the study design and population has been reported elsewhere (Brunekreef et al. 2002; Wijga et al. 2014). The present analysis included participants from this cohort with successful lung function measurements at 8 years of age; complete information on sex, age, height, and weight at the time of lung function measurement; and information on exposure to air pollution at the time of lung function measurement.

*Lung function measurements*. At age 8 years, all children of allergic mothers and a random sample of children of non-allergic mothers (*n* = 1,552) were invited for a medical examination including pulmonary function testing; of these, 1,058 children responded with a visit to one of the study hospitals. Children in the intervention and natural history groups were similar at age 8 years, and the intervention was shown not to have an effect on clinical outcomes (Gehring et al. 2012). In earlier work, we showed that combining these two groups did not affect associations between air pollution and lung function parameters (Gehring et al. 2013). A Jaeger pneumotachograph (Viasys Healthcare) was used for pulmonary function testing. We investigated the following lung function parameters: force expiratory volume in 1 sec (FEV_1_), forced vital capacity (FVC), and peak expiratory flow (PEF). Body weight and height were measured during the medical examination by trained research staff using calibrated equipment (Gehring et al. 2013).

*Air pollution exposure assessment*. We used a local dispersion and an LUR model to estimate annual average air pollution concentrations of NO_2_, PM_2.5_, PM_2.5_ soot, and PM_10_ at the participants’ home addresses at birth and at the time of the lung function tests.

LUR models were developed using measurement data from the ESCAPE (European Study of Cohorts for Air Pollution Effects) study collected during 2008–2011. In brief, three 2-week measurements within 1 year were conducted at 40 (PM) and 80 (NO_2_) locations, respectively, throughout the Netherlands. The measurements were temporally adjusted using data from a continuous regional reference site to generate annual average concentrations for LUR modeling. (For model structures and performances, see Supplemental Material, Table S1.) Details of the measurements and modeling efforts have been published elsewhere (Beelen et al. 2013; Cyrys et al. 2012; Eeftens et al. 2012a, 2012b). Detailed evaluations of model performances have been presented in a separate publication (Wang et al. 2013).
The Dutch dispersion model is a combination of a Gaussian plume model for the local scale and a Lagrangian trajectory model for long-distance transport (van Jaarsveld 2004), which produces estimates of background concentrations of NO_2_, PM_2.5_, PM_2.5_ soot, and PM_10_ with a spatial resolution of 1 × 1 km. Annual average air pollution levels at the current address were based on updated emission inventory data, actual meteorological parameters, and dispersion modeling (Velders and Diederen 2009). Background concentrations of PM_2.5_ soot were derived from fractions of primary PM_2.5_ in combustion emissions depending on the type of fuel (biomass, coal, oil, diesel, and petrol) as developed in the EUCAARI (European Integrated project on Aerosol, Cloud, Climate, and Air Quality Interactions) European research project (http://www.atm.helsinki.fi/eucaari/). Road traffic emissions were estimated by two standard Dutch models: “SRM1,” a street canyon model for inner urban roads, and “SRM2,” a line-source model for motorways. In SRM1, a source–receptor relationship has been specified as a function of the distance to the street axis for five different road types. SRM2 is based on a Gaussian plume model which takes into account vehicle-induced turbulence, the upwind roughness of the terrain, the presence of noise screens near the motorway, and atmospheric stability. Emission factors for road traffic of regulatory components (NO_x_/NO_2_, PM_2.5_, and PM_10_) are updated annually in the Netherlands, whereas for PM_2.5_ soot emission factors, fractions of primary PM_2.5_ exhaust emissions have been used for diesel- and petrol-fueled vehicles. More details about the applied dispersion models can be found elsewhere (Keuken et al. 2013; Wesseling 2003).

*Statistical analysis*. Pearson correlation coefficients were calculated to assess the agreement in estimated air pollution levels between different exposure modeling approaches and the agreement between the measured and dispersion-modeled predicted concentrations at the ESCAPE sites. Paired *t*-test was applied to investigate the differences between the means of the distributions estimated by the two different models.

We used linear regression analyses with natural log (ln)–transformed lung function parameters as the dependent variables to estimate associations between continuous lung function parameters and air pollution levels at the birth address and at the home address at the time of the lung function measurement, as described elsewhere (Gehring et al. 2013). For each pollutant we specified models adjusted for sex, ln(age), ln(weight), and ln(height) only; and fully adjusted models that also included the following individual-level variables: ethnicity; parental allergies; parental education; breastfeeding; maternal smoking during pregnancy; smoking, mold/dampness, and furry pets in the child’s home; and recent respiratory infections. We used fully adjusted models to compare associations with exposures estimated using the two different approaches. We also estimated associations using two-pollutant models that included both NO_2_ and PM_2.5_ estimated using either the dispersion model or the LUR model, to determine whether mutually adjusted effect estimates differed between the two exposure assessment methods. We estimated associations between air pollutants and lung function using fixed increments as used previously in the ESCAPE study (Gehring et al. 2013). These increments were 10 μg/m^3^ for NO_2_ and PM_10_, 1 × 10^–5^/m for PM_2.5_ soot, and 5 μg/m^3^ for PM_2.5_. Statistical significance was defined by a two-sided α-level ≤ 5%.

## Results

*Characteristics of the study population*. The studied population included 1,058 participants with an average age of 8 years and with 50.4% female (Table 1). Mean (± SD) FEV_1_, FVC, and PEF were 1.80 ± 0.25 L, 2.01 ± 0.30 L, and 3.79 ± 0.63 L/sec, respectively.

**Table 1:** Description of the study population and lung function measurements.

Variable	*n*	Percent or mean ± SD
Female sex	1,058	50.4
Respiratory infections	1,054	24.2
Allergic mother	1,058	66.1
Allergic father	1,055	33.3
Dutch ethnicity	1,044	95.7
High maternal SES	1,055	38.6
High paternal SES	1,043	42.9
Breastfeeding	1,058	52.6
Mother smoked during pregnancy	1,044	15.4
Smoking at child’s home^*a*^	990	15.7
Mold/dampness in child’s home^*a*^	985	28.8
Furry pets in home^*a*^	970	49.9
Height (cm)	1,058	132.90 ± 5.60
Weight (kg)	1,058	28.90 ± 4.80
Age (years)	1,058	8.10 ± 0.30
FEV_1_ (L)	1,058	1.80 ± 0.25
FVC (L)	1,058	2.01 ± 0.30
PEF (L/sec)	1,058	3.79 ± 0.63
^***a***^At the age of the lung function measurement.		

*Air pollution exposure*. Table 2 presents the distributions of estimated annual average concentrations of air pollutants by different exposure models for the area of the PIAMA cohort. Although *t*-tests indicated significant differences between mean estimates based on dispersion and LUR models for all of the pollutants (*p* < 0.01), mean values were similar. However, standard deviations (SDs) were larger for dispersion model estimates than estimates from the LUR models. PM_2.5_ soot concentrations were not directly comparable between LUR and dispersion models because each used different measurement techniques, with LUR estimates based on optical analysis reported as 10^–5^/m, and dispersion model estimates based on thermal analysis of elemental carbon reported as micrograms per cubic meter.

**Table 2:** Estimated annual average air pollution levels (*n* = 1,058).

Models	Mean ± SD	Minimum	P25	Median	P75	Maximum
NO_2_ (μg/m^3^)
Dispersion	23.0 ± 8.2	9.8	14.9	23.7	28.1	44.8
LUR	22.1 ± 6.3	9.4	17.5	22.4	26.2	52.1
PM_2.5_ (μg/m^3^)
Dispersion	15.9 ± 1.9	12.6	13.6	16.8	17.3	20.0
LUR	16.3 ± 0.6	14.9	15.6	16.5	16.7	19.3
PM_2.5_ soot^*a*^
Dispersion	0.7 ± 0.2	0.3	0.4	0.7	0.8	1.6
LUR	1.2 ± 0.2	0.9	1.0	1.2	1.3	2.1
PM_10_ (μg/m^3^)
Dispersion	23.8 ± 2.3	19.7	21.1	24.9	25.5	28.6
LUR	24.8 ± 1.0	23.7	24.0	24.5	25.1	29.8
P, percentile. ^***a***^PM_2.5_ soot was estimated by dispersion model using thermal detection method (μg/m^3^) and by LUR models using optical method (10^–5^/m).						

**Figure 1:**
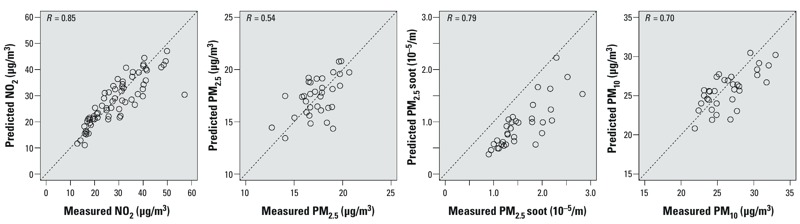
Pearson correlation coefficients of dispersion-modeled NO_2_ (*n *= 80), PM_2.5_, PM_2.5_ soot, PM_10_ (*n *= 40) with the same pollutants measured at the ESCAPE sites.

Performance evaluations of dispersion models with the measurements at the ESCAPE sites showed that the Pearson correlation coefficient was highest for NO_2_ (*R* = 0.85) and lowest for PM_2.5_ (*R* = 0.54) (Figure 1).

**Figure 2:**
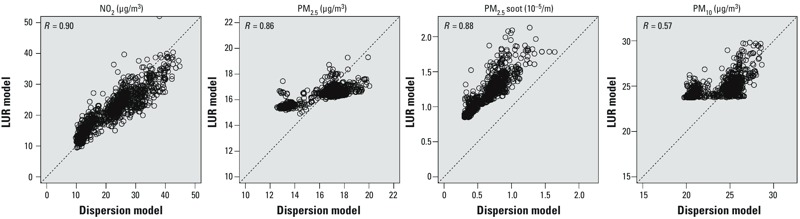
Pearson correlation coefficients of air pollution estimates between localized dispersion and LUR models at the PIAMA addresses (*n *= 1,058).

Figure 2 shows the scatter plots of the estimates between the dispersion and LUR models at the cohort addresses. Overall, the LUR model predictions correlated well with the estimates of the dispersion models for all the pollutants, except for PM_10_ (*R* = 0.57).

Table 3 shows strongest correlations of concentrations between any pair of pollutants by the dispersion model (*R* = 0.90–0.99), followed by the measurements (*R* = 0.75–0.93) and the LUR model (*R* = 0.63–0.91). The values in the correlation matrix of air pollution predicted by the LUR model (LUR panel in Table 3) were closest to the values in the correlation matrix between measured air pollutants (measured panel in Table 3).

**Table 3:** Pearson correlation coefficients between measured air pollution concentrations at the ESCAPE monitoring sites (NO_2_: *n *= 40; PM: *n *= 80) or modeled pollutants at PIAMA addresses (*n *= 1,058), respectively.

Models/pollutants	NO_2_	PM_2.5_	PM_2.5 _soot	PM_10_
Measured^*a*^
NO_2_	1
PM_2.5_	0.75	1
PM_2.5_ soot^*b*^	0.93	0.84	1
PM_10_	0.86	0.85	0.86	1
Dispersion
NO_2_	1
PM_2.5_	0.92	1
PM_2.5_ soot^*b*^	0.95	0.93	1	
PM_10_	0.90	0.99	0.92	1
LUR
NO_2_	1
PM_2.5_	0.75	1		
PM_2.5_ soot^*b*^	0.91	0.86	1
PM_10_	0.78	0.63	0.88	1
^***a***^Measured concentrations at the ESCAPE sites for LUR model development in the Netherlands. ^***b***^PM_2.5_ soot estimated by dispersion model using thermal detection method (μg/m^3^) and by LUR models using optical method (10^–5^/m).				

**Figure 3:**
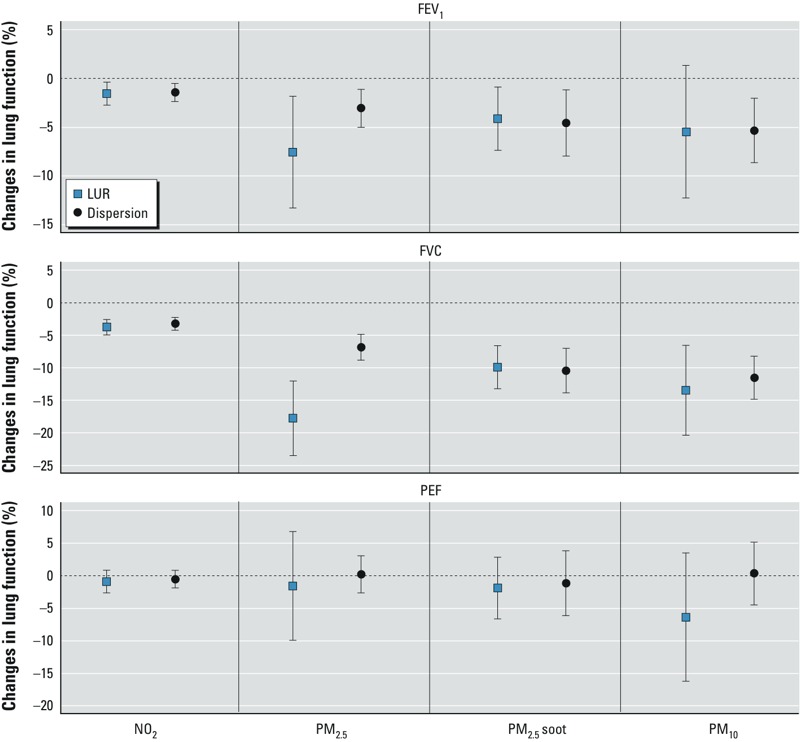
Adjusted associations of annual levels of air pollutants estimated by dispersion and LUR modeling approaches with FEV_1_, FVC, and PEF level (*n *= 1,058) at the PIAMA current addresses. The increment of each pollutant is calculated by 10 μg/m^3^ for NO_2_ and PM_10_, 1 × 10^–5^/m for PM_2.5_ soot, and 5 μg/m^3^ for PM_2.5_.

*Associations between lung function and exposure estimated by different approaches*. Overall, we found consistent negative associations between the lung function parameters FEV_1_ and FVC and long-term exposure to air pollution estimated by both dispersion and LUR models at the current home addresses (Figure 3). The magnitudes of the effect estimates were similar for NO_2_, PM_2.5_ soot, and PM_10_, but negative associations with PM_2.5_ were stronger for exposure estimates based on LUR compared with estimates based on dispersion modeling. The 95% confidence intervals (CIs) were similar for NO_2_ and PM_2.5_ soot but larger for PM_2.5_ and PM_10_ estimates by LUR models than for PM_2.5_ and PM_10_ estimates by dispersion models. No significant associations were found between air pollution estimated by any of the exposure approaches and PEF. Effect estimates for concentrations estimated at the birth addresses were somewhat weaker than for the current addresses (results not shown). Associations with FVC remained significant based on two-pollutant models for NO_2_ and PM_2.5_ when exposures were estimated using the localized LUR models (–2.4% difference; 95% CI: –4.1, –0.8 and –9.5% difference; 95% CI: –18.2, –0.9 for NO_2_ and PM_2.5_, respectively) but were no longer significant when exposures were estimated using the dispersion models (–2.0% difference; 95% CI: –4.2, 0.3 and –3.0% difference; 95% CI: –7.8, 2.0, respectively).

## Discussion

Model predictions of LUR and dispersion for PM_2.5_, NO_2_, and PM_2.5_ soot correlated very well. For PM_10_ correlations between LUR and dispersion models were more moderate. For PM_2.5_ the variability in concentrations predicted by the LUR model was smaller than for the dispersion model, whereas for NO_2_ and PM_2.5_ soot, variability was similar between the two models. LUR and dispersion predictions for PM_2.5_ soot are expressed in different units (PM_2.5_ absorbance in 10^–5^/m in the LUR model and micrograms per cubic meter in the dispersion model). If the average conversion factor in a recent review (Janssen et al. 2011) is applied (1 unit absorbance = 1.1 μg/m^3^ elemental carbon), the dispersion and LUR models predict slightly different absolute levels. The better agreement for NO_2_ compared with PM mass agrees with a recent comparison between dispersion and LUR models (de Hoogh et al. 2014). The explanation offered by the authors was that both methods perform better for traffic-related pollutants than for other pollutants, when appropriate input data are available. This interpretation is supported by our results for PM_2.5_ soot, which was not evaluated in the previous paper (de Hoogh et al. 2014) and is strongly affected by traffic emission in the Netherlands.

Previous studies have looked at correlations between LUR and dispersion modeled concentrations of NO_2_ (Beelen et al. 2010; Cyrys et al. 2005; Marshall et al. 2008). Only the study by Cyrys et al. (2005) documented a reasonably high Pearson correlation coefficient of 0.83 between the two models. The Pearson correlation coefficient of 0.90 that we found compares favorably with this study as well as with a recent multicenter study published by de Hoogh et al. (2014), who found a median Pearson correlation coefficient of 0.75 in 13 different European study areas. The Pearson correlation coefficient of 0.86 that we found for PM_2.5_ was higher than the median Pearson correlation coefficient of 0.28 in de Hoogh et al. (2014), and our correlation for PM_10_ of 0.57 was also higher than the correlation of 0.39 in that paper.

*Comparison of the effect estimates of the association between long-term exposure to air pollution and lung function in children using LUR and dispersion models.* This study shows that different exposure approaches revealed generally similar estimates of the association between long-term exposure to NO_2_ and PM_2.5_ soot and lung function in a Dutch birth cohort. Effect estimates for PM_2.5_ and PM_10_ were larger for the LUR estimates than for the dispersion estimates, but with wider confidence intervals. One explanation could be that the PM_2.5_ and PM_10_ dispersion models did not predict the measured spatial variation of PM_2.5_ and PM_10_ well (Figure 1). However, effect estimates were expressed over fixed concentration ranges. The dispersion models predicted wider concentration ranges for PM_2.5_ and PM_10_ than did the LUR models, and as a consequence the 95% CIs of the LUR-modeled effect estimates were larger than those of the dispersion-modeled effect estimates.

A strength of our study relates to the comparisons for PM_2.5_ and PM_2.5_ soot in addition to NO_2_ and PM_10_. Previous studies based on dispersion models focused primarily on NO_2_ and PM_10_ health effects (Downs et al. 2007; Jacquemin et al. 2013; Schultz et al. 2012; Sellier et al. 2014), with only one exception for PM_2.5_ in Oslo (Oftedal et al. 2008). Moreover, our study employed well-validated Dutch dispersion and LUR models with fine spatial resolution and reliable predictions of air pollution levels.

We estimated effects of a similar magnitude on lung function for all the strongly correlated air pollutants assessed by the dispersion models (Pearson correlation coefficients: 0.92–0.99; Table 3), probably because the sources are assumed to be largely the same: The dispersion model used presumed fractions of PM emission factors derived from exhaust emissions and applied a scaling approach to estimate the PM metrics. In contrast, correlations between the air pollutants were weaker when estimated using the localized LUR models, and very similar to corresponding correlations between measured air pollutant concentrations, because the LUR input data came from real measurements. Predictor variables in the LUR models frequently included population (or residence) density, a surrogate for sum of household activities (e.g., cooking and heating emissions) that were absent in the emission inventory for the dispersion modeling. Two-pollutant models with NO_2_ and PM_2.5_ indicated more robust and independent effects of individual pollutants on FVC using the exposure estimates from the LUR models than from the dispersion models.

A limitation of this study is that we do not know how generalizable the findings of our analysis are to other cities and areas. We acknowledge that both dispersion and LUR models might produce exposure misclassifications, and the degree of the impact depends on a variety of factors differentiating across geographical locations. For dispersion models, potential measurement errors may be affected by local emission inventory, the method of air pollution simulation, and the spatial resolution of grid cells. For LUR models, validity depends on the number of sampling sites, the quality of GIS variables, and the modeling procedures (Basagaña et al. 2012).

In summary, LUR and dispersion model predictions for PM_2.5_, NO_2_, and PM_2.5_ soot were very well correlated (Pearson correlations, 0.86–0.90). For PM_10_, correlations between LUR and dispersion models were more moderate. Health effect estimates did not depend on the type of model used to estimate exposure in the study population of Dutch children.

## Supplemental Material

(120 KB) PDF
