# Supplementary material for: Air Pollution and Lung Function in Dutch Children: A Comparison of Exposure Estimates and Associations Based on Land Use Regression and Dispersion Exposure Modeling Approaches
Source: Environ Health Perspect. 2015 Apr 3;123(8):847–51. doi: 10.1289/ehp.1408541 (PMC4529005; doi:10.1289/ehp.1408541)
Supplement: (120 KB) PDF [file ehp.1408541.s001.acco.pdf]

*Environ Health Perspect*
doi: 10.1289/ehp.1408541

**Note to Readers:** *EHP* strives to ensure that all journal content is accessible to all readers. However, some figures and Supplemental Material published in *EHP* articles may not conform to 508 standards due to the complexity of the information being presented. If you need assistance accessing journal content, please contact ehp508@niehs.nih.gov. Our staff will work with you to assess and meet your accessibility needs within 3 working days.

# Supplemental Material

# Air Pollution and Lung Function in Dutch Children: A Comparison of Exposure Estimates and Associations Based on Land Use Regression and Dispersion Exposure Modeling Approaches

Meng Wang, Ulrike Gehring, Gerard Hoek, Menno Keuken, Sander Jonkers, Rob Beelen, Marloes Eeftens, Dirkje S. Postma, and Bert Brunekreef

## Table of Contents

**Table S1.** LUR model structures and performances [leave-out-out-cross-validation (LOOCV) $R^2$ and root mean square error (RMSE)] used in this study.

**Table S1.** LUR model structures and performances [leave-out-out-cross-validation (LOOCV) $R^2$ and root mean square error (RMSE)] used in this study.

| Pollutants | LOOCV $R^{2a}$ | RMSE[b] | Predictor variables in LUR models |
|---|---|---|---|
| $NO_2$ | 0.81 | 5.1 | Regional background $NO_2$, population density in 5000m buffer, traffic load in 50m buffer, road length in 1000m buffer, heavy traffic load in 25m and 25 to 500m buffer, inverse distance to the nearest road. |
| $PM_{2.5}$ | 0.61 | 1.2 | Regional background $PM_{2.5}$, major road length in 50m buffer, traffic load at major road in 1000m buffer. |
| $PM_{2.5}$ soot | 0.89 | 0.2 | Traffic load in 500m buffer, major road length in 50m buffer, regional background $PM_{2.5}$ soot, high and low residence density in 5000m buffer, heavy traffic load in 50m buffer. |
| $PM_{10}$ | 0.60 | 2.3 | Traffic load at major road in 500m buffer, population density in 5000m buffer, major road length in 50m buffer. |

[a]LOOCV $R^2$: leave-one-out-cross-validation $R^2$. [b]RMSE: root mean squared error from leave-one-out-cross-validation.
